# A JSBSim Sensor-Interface Protocol for Selecting Learned Fixed-Wing Flight-Dynamics Surrogates

**DOI:** 10.3390/s26154888

**Published:** 2026-08-03

**Authors:** Yihao Feng, Jun Li, Sen Yang, Yulong Ji

**Affiliations:** 1 School of Aeronautics and Astronautics, Sichuan University, Chengdu 610065, China; 2 System of Systems and Artificial Intelligence Key Laboratory of Sichuan Province, Chengdu 610091, China; 3 AVIC Chengdu Aircraft Design and Research Institute, Chengdu 610091, China

**Keywords:** learned dynamics models, autonomous flight, onboard sensing, state estimation, rollout evaluation and selection framework, task-conditioned surrogate selection, model-based planning

## Abstract

Autonomous flight planners consume sensor-derived estimated states rather than simulator ground truth. Learned dynamics surrogates are queried over a planning horizon through architecture-native multi-step interfaces, so strong short-horizon accuracy can still yield unreliable long-horizon trajectory scores. We address this with a JSBSim sensor-interface evaluation protocol that assesses surrogate predictions from onboard sensing and lightweight estimation—inertial measurement unit (IMU), global navigation satellite system (GNSS)/air-data, barometric, and vertical-speed channels with delay, dropout, asynchronous refresh, and estimator filtering—and guides surrogate selection by a planning task. This paper contributes an evaluation-and-selection methodology for learned flight-dynamics surrogates used by autonomous planners; it is not a new flight-dynamics, guidance, or control method, and it does not propose a new neural architecture. The contribution is a transparent and auditable sensor-interface and estimator-conditioned protocol, instantiated on five surrogate families over a 20 s prediction horizon. We emphasize at the outset that under strict native-unit physical tolerances, all evaluated surrogates diverge on 90–100% of validation windows and none is field-ready; all results are comparative JSBSim stress-test evidence, not field-readiness evidence. Results reveal criterion-dependent ordering: long short-term memory (LSTM) networks are strongest on root-mean-square error (RMSE@1s/RMSE@20s), shared-threshold failure, and absolute fidelity, whereas the Transformer is favored under self-scaled divergence and capped-risk criteria. These aggregate scores combine heterogeneous simulator-coordinate units and are benchmark diagnostics rather than physical safety margins. Because different threshold families select different winners, our central conclusion is not that one surrogate is best, but that the choice of metric and threshold family is itself part of the benchmark assessment. In separately generated JSBSim candidate-screening episodes, Transformer has approximately 8% lower mean cost under abstract sensing corruption, whereas estimator-loop screening shifts toward LSTM; feedback-rich tracking remains exploratory at 40 episodes per scenario and has mixed cost and success endpoints. Cross-aircraft transfer degrades substantially. Surrogates should be assessed using sensing-aware, estimator-conditioned, task-specific long-horizon criteria, not short-horizon accuracy alone.

## 1. Introduction

Autonomous flight planners operate on sensor-derived estimated states rather than simulator ground truth. Therefore, the reliability of learned dynamics surrogates depends on how sensing noise, estimator filtering, delay, dropout, and asynchronous refresh affect long-horizon rollout. This makes surrogate selection a sensor-driven planning problem, not only a model-accuracy comparison. We emphasize at the outset that under strict native-unit physical tolerances, all evaluated surrogates diverge on 90–100% of rollouts, and none is field-ready; all results in this paper are comparative stress-test evidence for model selection within JSBSim fixed-wing benchmarks, not evidence of operational readiness.

Problem statement. We do not seek a single surrogate that is best for every planning use; on this benchmark, no such universal winner exists (Section 4). Instead, we ask how a transparent, auditable task- and estimator-conditioned heuristic can organize multiple long-horizon evaluation views into a documented surrogate choice. The priority tuples formalized later in Section 3 are retrospective, benchmark-specific summaries informed by the reported analyses; they are not preregistered or independently held-out selector validation. Candidate-screening cost is treated descriptively, and feedback-rich tracking remains exploratory with mixed cost and success endpoints.

This JSBSim study evaluates how the planner-facing state produced by onboard sensing and estimation affects learned flight-dynamics surrogates used in autonomous planning. Inertial measurement unit (IMU), global navigation satellite system (GNSS)/air-data, barometric, and vertical-speed channels define that interface through their noise, timing, dropout, and filtering characteristics. The study does not propose a new physical sensor; it evaluates the measurement-to-planner interface using five surrogate families as the comparison set.

Model-predictive control (MPC) and model-based learning for autonomous flight require evaluating many candidate trajectories inside an inner optimization loop. JSBSim [1] provides an open-source fixed-wing flight-dynamics simulation framework; we observe in our benchmark setup that its central-processing-unit (CPU)-oriented step execution makes batched rollout evaluation expensive in surrogate-comparison workloads. A learned surrogate can enable batched graphics-processing-unit (GPU) prediction for trajectory scoring or policy improvement, but low short-horizon prediction error is not enough: the surrogate must remain usable over the full planning horizon from the planner-facing estimated state.

In practical autonomous flight systems, this estimated state comes from onboard sensing and state-estimation pipelines, including IMU-derived attitude and angular-rate estimates, air-data and navigation-derived velocity information, and altitude or climb information from barometric and navigation sources. Therefore, the planning-time reliability of a learned dynamics surrogate depends not only on nominal predictive accuracy, but also on whether its long-horizon rollout remains usable when planning is driven by measured or estimated flight states.

This matters because planning operates on multi-step trajectories, not isolated transitions. A model that fits local dynamics well can still produce unusable 5–20 s predictions; for recursive MLP/GRU/LSTM models this can include feedback accumulation, whereas the TCN and Transformer use direct sequence-style prediction interfaces. The practical question is not only which architecture predicts most accurately, but which learned dynamics model generates rollouts that remain usable for downstream flight decision-making in a sensing-to-estimation-to-planning chain.

**Figure 1:**
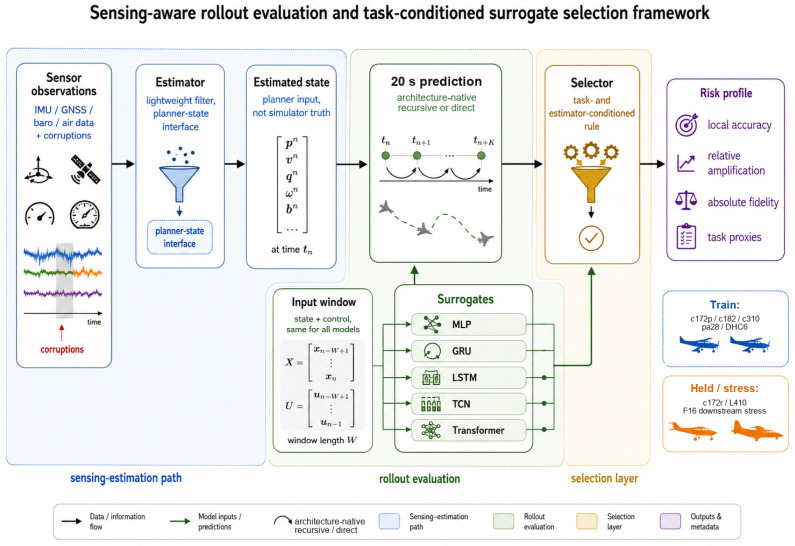
Protocol overview for sensing-aware rollout evaluation and task-conditioned surrogate selection. Sensor observations are converted by a lightweight estimator into the planner-facing state; candidate surrogates are then evaluated through their architecture-native 20 s prediction interfaces from that estimated state. The protocol reports a multi-view rollout-risk profile spanning local accuracy, self-scaled relative amplification, absolute fidelity, and task proxies, and also includes transfer to held-out aircraft types. Abbreviations: IMU, inertial measurement unit; GNSS, global navigation satellite system; MLP, multilayer perceptron; GRU, gated recurrent unit; LSTM, long short-term memory; TCN, temporal convolutional network.

We therefore propose a sensing-aware evaluation protocol that couples architecture-native 20 s prediction with controlled sensing corruption, estimated-state inputs, and an aircraft-specific estimator-loop stress test. It summarizes each candidate by local accuracy, self-scaled amplification, capped risk, native-unit fidelity, and directly simulated downstream outcomes, then organizes those views by planning task and estimator path. The comparison set comprises a multilayer perceptron (MLP), gated recurrent unit (GRU), long short-term memory (LSTM), temporal convolutional network (TCN), and Transformer; the lightweight sensing model isolates planner-interface effects rather than emulating a certified navigation system. Figure 1 summarizes the protocol.

The main contributions of this paper are:

1. We formulate surrogate assessment as a sensor-interface-conditioned problem and evaluate five model families through their implemented 20 s interfaces—recursive for MLP/GRU/LSTM and direct sequence-style for TCN/Transformer—under controlled sensing and estimation conditions.
2. We combine local error, self-scaled amplification, capped risk, and native-unit fidelity, showing that metric and threshold family change the benchmark ordering: LSTM is favored by shared-threshold and absolute-fidelity views, whereas Transformer is favored by self-scaled and capped-risk views.
3. We connect these views to directly simulated screening and exploratory tracking, yielding a transparent task- and estimator-conditioned heuristic whose scope and statistical limitations are reported explicitly.

The contribution is the documented coupling of sensing/estimation, architecture-native long-horizon prediction, threshold-family sensitivity, and planner-facing task checks; it is not a new network, flight-dynamics model, guidance law, or control method. Claims are restricted to the stated JSBSim suites: transfer degrades substantially, tracking remains exploratory, and the auxiliary logged-control inclusion diagnostic is not used as ranking-fidelity evidence.

### Sensor-Interface Contribution and Scope

Table 1 maps the sensor-to-planner interface to its uncertainty mechanism, planner-state effect, and evaluation view.

## 2. Related Work

### 2.1. Learned Dynamics for Planning and Control

Model-based reinforcement learning (RL) and planning methods learn dynamics to reduce sampling or simulation cost [2,3,4,5], but they often limit the learned-model rollout horizon and mitigate model error through short rollouts, ensembles, or conservative operational strategies. Ensemble-based methods such as probabilistic ensembles with trajectory sampling (PETS) [2] propagate predictive uncertainty to detect when the model extrapolates beyond its training distribution, complementing our rollout-aware selection with uncertainty quantification. Pessimistic model-based methods such as model-based offline policy optimization (MOPO) [6] and model-based offline reinforcement learning (MOReL) [7] penalize rollouts that enter high-uncertainty regions, using capped or penalized returns to avoid overoptimistic planning. Our capped RMSE metric shares the spirit of these penalty-based approaches, but differs in two key respects: (1) we apply the penalty at evaluation time to compare candidate surrogates rather than during policy optimization, and (2) we explicitly condition the evaluation on the sensing–estimation interface, which MOPO/MOREL do not address. Our focus is therefore complementary: when a learned model is used through its architecture-native multi-step interface inside flight planning under sensing-supported estimated states, how should it be selected for long-horizon usability? Prior work has already noted that short-horizon prediction quality and rollout usefulness can diverge [8,9,10]. We operationalize that concern for fixed-wing flight dynamics and connect it directly to sensing-conditioned model selection.

### 2.2. Flight-Dynamics Modeling and Identification

Classical aircraft identification relies heavily on parametric flight-dynamics models [11], which provide physically structured dynamics but do not directly address learned-surrogate selection under long-horizon planner use. Neural flight-dynamics models [12,13] are directly relevant; they evaluate predictive accuracy over task-specific horizons, but do not focus on architecture-native long-horizon usability under sensing-conditioned planner inputs. Recent aerospace-control studies have used learned models directly inside flight-control and optimization loops, including sparse online Gaussian-process adaptation for aircraft control and sparse neural dynamics embedded in nonlinear model-predictive control for fixed-wing trajectory tracking [14,15]. These studies optimize a particular learned model or controller, whereas the present work evaluates how candidate surrogate families should be compared under a sensor-derived planner interface and multiple long-horizon metric families. That literature rarely asks whether the most accurate local predictor is also the most reliable model for long-horizon planning rollout, candidate screening, or measured-state operational evaluation.

### 2.3. Sensing, Estimation, and Onboard State Propagation

In autonomous flight systems, planning modules operate on states produced by sensing and estimation pipelines rather than on perfect simulator truth. This connects our problem to onboard sensing and state-estimation research, where IMU, navigation, and air-data fusion determine the state available to guidance and control. Multi-sensor fusion for unmanned aerial vehicle (UAV) and micro aerial vehicle (MAV) state estimation commonly combines IMU propagation with lower-rate complementary measurements such as GNSS, barometer, magnetometer, or vision in extended Kalman filter (EKF)-style pipelines [16,17,18]. Recent sensing papers also emphasize that multi-rate sensing and delayed observations materially change estimator design and downstream state quality [19]. Recent UAV navigation studies further show that resilient multi-sensor fusion, learned global navigation satellite system/inertial navigation system (GNSS/INS) outage compensation, and real-time kinematic GNSS (RTK-GNSS)/microelectromechanical-system IMU (MEMS-IMU) integration materially reshape the state estimates available to onboard autonomy [20,21,22]. These results reinforce that the sensing-estimation pipeline feeding a planner is an actively engineered and imperfect interface rather than a fixed ground-truth channel. Recent surveys of UAV sensor integration further highlight that robust autonomy depends on how sensing, communication, and onboard inference are coupled in operational environments [23]. Our contribution is a sensor- and estimator-conditioned evaluation-and-selection protocol that reveals how surrogate-model choice shifts when the planning context is treated as measured or estimated state, rather than idealized simulator truth. Unlike UAV state-estimation studies that design fusion filters or improve navigation accuracy, we hold the estimator interface explicit and ask how its output changes downstream learned-surrogate selection. The distinction from a conventional accuracy benchmark is the coupling itself: prior learned-dynamics comparisons rank architectures by short-horizon prediction error on clean states, and prior sensor-fusion and model-based-planning work each address a single stage in isolation. The proposed protocol instead connects estimator-conditioned architecture-native long-horizon prediction, task-specific evaluation views, threshold-family sensitivity, and downstream planner-facing screening and tracking checks in one documented benchmark procedure. The reported question is therefore not only “which model predicts most accurately” but “which evaluation view is relevant for a sensing-aware planner, and how does that view change with the threshold family and estimator path?”

### 2.4. State-Estimation-Aware Planning

Sensor-driven autonomy also requires asking how state-estimation errors propagate into planning-time prediction. Noise, filtering lag, dropout, and asynchronous refresh do not only perturb the current state estimate; once a learned dynamics model is queried over a long horizon, they can change the trajectory distribution used for scoring, screening, or feedback-rich tracking. Recent model-learning work similarly shows that sensing quality can directly affect the learned model used for control or planning [24]. This paper targets that interface between sensing, estimation, and learned surrogate rollout by evaluating models on the planner-facing estimated state rather than on clean simulator state alone.

### 2.5. Sequence Architectures and Long-Horizon Forecasting

Recurrent networks, TCNs, and Transformers are established sequence-modeling architectures, and TCN/Transformer variants have been evaluated for long-sequence forecasting [25,26,27]. Comparisons among these families are usually framed by short-horizon forecasting error. Our protocol uses a different criterion: whether architecture-native long-horizon prediction remains bounded and decision-relevant over the planner horizon, and whether that conclusion changes once the prediction input is treated as a sensing-supported onboard state.

## 3. Problem Setting and Sensing-Aware Evaluation–Selection Framework

### 3.1. Planning-Time Rollout Setting

We model fixed-wing aircraft dynamics as a controlled discrete-time system with state $s_{t}\in R^{11}$ and control $a_{t}\in R^{4}$. Given a context window of past states and future controls, a learned surrogate predicts an *H*-step state sequence:

(1) $${\hat{s}}_{t+1:t+H}=f_{\theta }\;\left({\hat{s}}_{t-T_{c}+1:t},\;a_{t+1:t+H}\right),$$

Equation (1) uses $T_{c}=50$ context steps (5 s at 10 Hz, chosen to match the receding-horizon window used in the downstream MPC tracking loop). The implemented families share this input/output interface but not one common rollout mechanism. MLP, GRU, and LSTM recursively feed predicted states into subsequent steps; TCN predicts a sequence of future deltas and cumulatively integrates them, while Transformer directly predicts the future residual sequence from the context and future controls. Accordingly, this paper compares architecture-native long-horizon prediction behavior and does not attribute every model’s error growth to recursive state feedback.

### 3.2. Sensing Context

Although our benchmark is generated in simulation, the state variables are chosen to match quantities that, in an onboard system, are supplied by sensing and estimation modules. Attitude and angular-rate channels correspond to IMU-driven attitude estimation, while speed, altitude, and vertical-motion channels correspond to air-data, barometric, and navigation-estimation outputs. Accordingly, the rollout input should be interpreted as a measured or estimated flight state rather than perfect simulator ground truth. This distinction matters because a surrogate with acceptable one-step fit can still amplify sensing or estimation imperfections over long horizons, degrading the reliability of downstream planning. Formally, we treat the planner-facing state as

(2) $$\begin{array}{cc} y_{t} & \;=h\left(s_{t}\right)+n_{t}+b_{t},\\ {\hat{s}}_{t} & \;=E_{\mathrm{est}}\left(y_{1:t},a_{1:t}\right), \end{array}$$

In Equation (2), $s_{t}$ is the latent simulator state, $y_{t}$ is the sensed vector, $n_{t}$ is measurement noise, $b_{t}$ is a slowly varying bias or drift vector, and $E_{\mathrm{est}}$ denotes the onboard estimator function, not a mathematical expectation operator. Accordingly, the surrogate mapping in Equation (1) is evaluated using the planner-facing history ${\hat{s}}_{t-T_{c}+1:t}$, so the model-selection question is conditioned on ${\hat{s}}_{t}$, not on idealized $s_{t}$.

### 3.3. Aircraft-Specific Estimator-in-the-Loop Setting

We instantiate (2) with an aircraft-specific IMU/GNSS/baro observation model and a lightweight complementary/Kalman-style estimator. Angular rates are measured by a gyro channel and propagated as

(3) $$\begin{array}{cc} {\hat{\eta }}_{t}^{-} & \;=\mathrm{wrap}\;\left({\hat{\eta }}_{t-1}+\Delta t\;y_{t}^{\omega }\right),\\ {\hat{\eta }}_{t} & \;=\mathrm{wrap}\;\left[{\hat{\eta }}_{t}^{-}+\left(1-\beta \right)\mathrm{wrap}\;\left(y_{t}^{\eta }-{\hat{\eta }}_{t}^{-}\right)\right], \end{array}$$

In Equation (3), $\eta ={\left[\phi ,\theta ,\psi \right]}^{⊤}$ and β∈[0.95,0.97] is the complementary gain. Velocity, altitude, and climb-rate channels use scalar innovation updates:

(4) $$\begin{array}{cc} {\hat{v}}_{t} & \;={\hat{v}}_{t-1}+K_{v}\;\left(y_{t}^{\mathrm{gnss}}-{\hat{v}}_{t-1}\right),\\ {\hat{h}}_{t} & \;={\hat{h}}_{t}^{-}+K_{h}\;\left(y_{t}^{\mathrm{baro}}-{\hat{h}}_{t}^{-}\right),\\ {\hat{\dot{h}}}_{t} & \;={\hat{\dot{h}}}_{t-1}+K_{\dot{h}}\;\left(y_{t}^{\mathrm{vario}}-{\hat{\dot{h}}}_{t-1}\right), \end{array}$$

Equation (4) uses ${\hat{h}}_{t}^{-}={\hat{h}}_{t-1}+\Delta t\;{\hat{\dot{h}}}_{t-1}$. The estimator is intentionally lightweight to isolate the effect of planner-facing state uncertainty, while explicitly inserting a sensor-to-estimator-to-planner interface into the benchmark. Its structure is consistent with prior flight-state estimators that fuse IMU propagation with lower-rate complementary measurements and explicitly handle multi-rate or delayed sensing [16,17,18,19]. The purpose of this sensing model is not to emulate a certified avionics-grade navigation system, but to expose how planner-facing state uncertainty changes surrogate rollout behavior and model selection. The estimator is not intended to benchmark navigation-filter performance. It is used as a controlled interface that converts sensor-level corruption into planner-facing state estimates, allowing the same candidate surrogates to be compared under a consistent sensing-estimation path. A lightweight estimator is adequate because the claimed scope is comparative surrogate selection, not certified navigation: every candidate is compared under the same fixed estimator path, so the selection outcome depends on relative surrogate behavior rather than on the absolute accuracy of the filter. All selection claims are accordingly conditioned on this fixed lightweight estimator path and do not assert certified navigation performance; integrating a full extended/unscented Kalman filter (EKF/UKF) navigation stack is left to future work.

Table 2 summarizes the sensor and estimator settings used in the estimator-in-the-loop experiment.

### 3.4. Sensing-Aware Rollout Evaluation and Task-Conditioned Selection Framework

The proposed protocol is an evaluation-and-selection framework rather than a new surrogate architecture. Its role is to test candidate learned dynamics models under the planner-facing sensing–estimation interface and organize the resulting evidence for a target planning task within JSBSim benchmark scenarios. Given a candidate set of surrogates and a target planning setting, the workflow is summarized in Algorithm 1.
**Algorithm 1** Sensing-Aware Rollout Evaluation and Task-Conditioned Surrogate Selection**Input:** Candidate surrogate set F, planner-facing sensing–estimation interface, target planning task, rollout horizon *H*.**Output:** Task-conditioned surrogate choice.**Step** **1:**Construct the *planner-facing state* using measured or estimated channels rather than simulator truth, as formalized by Equations (1) and (2).**Step** **2:**Generate operationally relevant sensing conditions, including measurement noise, filtered estimated-state input, delay, dropout, asynchronous refresh, and, when available, an explicit estimator-in-the-loop interface.**Step** **3:**Query each surrogate over the full planning horizon (H=20 s in this benchmark) through its implemented architecture-native interface: recursive for MLP/GRU/LSTM and direct sequence-style for TCN/Transformer.**Step** **4:**Evaluate local accuracy (short-horizon aggregate RMSE), self-scaled long-horizon error growth (divergence rate), absolute fidelity (shared aggregate-threshold and physical native-unit views), and downstream task proxies (screening and tracking behavior).**Step** **5:**Organize the metrics for the target task using the transparent benchmark-specific heuristic defined below: screening emphasizes observed task cost and long-horizon risk, whereas state-faithful prediction and feedback-rich control emphasize absolute-error fidelity and controller-facing assessment.

In plain language, the protocol runs five steps before it reports a task-conditioned benchmark choice. Step 1 builds the planner-facing state from measured or estimated sensor channels instead of simulator truth. Step 2 applies measurement noise, estimator filtering, delay, dropout, asynchronous refresh, and, where available, an explicit estimator-in-the-loop path. Step 3 evaluates each candidate through its implemented 20 s prediction interface; MLP/GRU/LSTM are recursive, while TCN/Transformer generate future sequences directly. Step 4 scores local accuracy, self-scaled long-horizon error growth, absolute fidelity, and downstream screening/tracking proxies rather than collapsing them into one number. Step 5 organizes these views using the transparent retrospective priority order formalized below. The remainder of this section formalizes each step.

Formally, Algorithm 1 produces a task-conditioned selection operator. For a candidate surrogate f∈F, let

(5) $$r\left(f\right)=\left[m_{\mathrm{local}}\left(f\right),\;m_{\mathrm{rel}}\left(f\right),\;m_{\mathrm{cap}}\left(f\right),\;m_{\mathrm{abs}}\left(f\right),\;m_{\mathrm{task}}\left(f\right),\;m_{\mathrm{energy}}\left(f\right)\right]$$

Equation (5) denotes the multi-view rollout-risk profile under the planner-facing estimated-state interface. The entries correspond to local accuracy, self-scaled relative amplification, capped RMSE, physical absolute-error fidelity, directly simulated downstream task cost, and a scale-normalized energy-state composite (forward speed, altitude, and vertical speed), respectively. The entries are scalar summaries drawn from the reported open-loop and downstream benchmark suites; lower values are better. Table 3 gives the explicit definition of each metric.

The implementation computes $m_{\mathrm{local}}$, $m_{\mathrm{rel}}$, and $m_{\mathrm{cap}}$ after mapping predictions back to the simulator-output coordinate vector. Because that vector contains heterogeneous units, these three quantities are benchmark aggregate scores rather than dimensional physical-error measures; $m_{\mathrm{abs}}$ and the reported channel-wise RMSE values provide the native-unit interpretation. For a planning task τ and estimator path *e*, we define

(6) $$S_{\tau ,e}\left(F\right)=\arg\min_{f\in F}\Phi_{\tau ,e}\;\left(r(f)\right),$$

Equation (6) defines the task-conditioned choice, where $\Phi_{\tau ,e}$ is the lexicographic priority rule in Table 4. The operator is a deterministic summary of the benchmark metrics reported in this study; because downstream task metrics are drawn from the available proxy suites, it is not an independently held-out selector-validation procedure. Ties within one percentage point for rate metrics or within one standard error for continuous costs are broken by the next metric in the listed order; if the tie persists, the lower-latency model is selected. The framework therefore outputs $S_{\tau ,e}\left(F\right)$, a task- and estimator-conditioned model choice, rather than a universal winner. We emphasize that $\Phi_{\tau ,e}$ is a transparent, benchmark-specific audit heuristic, not a newly proposed optimization or selection algorithm and not a preregistered selector. Its value is to expose the metric priorities explicitly so that the resulting choice is documented and auditable on the reported benchmark, while downstream results are interpreted as descriptive consistency checks rather than independent validation. The five-model comparison used in this paper is the empirical instantiation of this framework.

The priority tuples in Table 4 summarize the observed metric–task relationships in the available benchmark data. Surrogates are fitted using the training split; open-loop metric settings are summarized on validation windows; and screening/tracking use separately generated JSBSim task episodes. The tuple assessment is therefore transparent and documented but retrospective rather than an untouched-test validation claim.

This explicit operator makes disagreements auditable: if a model selected by a reported tuple performs worse on the corresponding task proxy than alternatives emphasized by lower-priority metrics, that mismatch identifies a limitation of the benchmark heuristic rather than being hidden by a single aggregate score. It also separates diagnostic views from selection views. Self-scaled relative divergence is one diagnostic for abstract candidate screening, but it cannot override fixed physical or energy-state errors for state-faithful prediction, feedback-rich tracking, or estimator-loop assessment.

Rationale for the priority order. The tuples are organized retrospectively from the metric–task associations reported in Section 4. For abstract screening, the observed JSBSim screening task cost is placed first, followed by capped and self-scaled long-horizon risk. Under the aircraft-specific estimator loop, screening task cost remains first and the scale-normalized energy-state composite is added as a guardrail because the observed screening ordering follows the native-unit channel errors. State-faithful prediction and cross-aircraft transfer assessment emphasize $m_{\mathrm{abs}}$, while tracking retains both absolute fidelity and task cost because its endpoints are mixed. These priorities are an explicit benchmark-specific synthesis for interpretation and hypothesis generation, not evidence of a preregistered selector or an independently replicated operational rule.

### 3.5. Relative Rollout Divergence

Short-horizon RMSE already measures local fit, so our relative stability metric focuses on whether error grows relative to each model’s own short-horizon scale over the implemented long-horizon prediction interface. Let d=11 and let $e_{i,t,j}={\hat{s}}_{t,j}^{\left(i\right)}-s_{t,j}^{\left(i\right)}$ denote the error after predictions and targets are mapped back to the simulator-output coordinate vector. The implementation defines the model’s aggregate root-mean-square error over the first 1 s horizon (10 steps) as

(7) $$\epsilon_{1s}=\sqrt{\frac{1}{10Nd}\sum_{i=1}^{N}\sum_{t=1}^{10}\sum_{j=1}^{d}{\left(e_{i,t,j}\right)}^{2}},$$

where the coordinates retain their simulator units. This aggregate is therefore a defined benchmark score over a heterogeneous coordinate vector, not a single physical quantity with one unit. For multiplier α, a rollout is marked as diverged if

(8) $$\max_{1\leq t\leq 200,\;1\leq j\leq d}\left|e_{i,t,j}\right|>\alpha \epsilon_{1s},$$

i.e., the largest componentwise simulator-coordinate error at any point in the 200-step rollout exceeds α times the model’s own short-horizon error scale. The main tables use α=5 as the nominal midpoint of the reported sensitivity set α∈{3,5,7}. The same multiplier is applied to every model and sensing condition, and we report the full sweep so that no conclusion depends on the nominal multiplier alone. The *divergence rate* is the fraction of evaluation rollouts that diverge. This criterion asks whether long-horizon error becomes several times larger than the model’s own short-horizon error scale. It should therefore be read as a self-scaled benchmark amplification metric, not as a claim that the selected model has the smallest absolute long-horizon error.

Important limitation. The self-scaled threshold $\alpha \epsilon_{1s}$ could mechanically favor models with higher $\epsilon_{1s}$ by giving them a looser bound. In addition, both the bound and the componentwise maximum are computed on a heterogeneous simulator-coordinate vector, so this score is not dimensionless and should not be interpreted as a physical safety margin. We expose rather than hide these limitations by reporting multiple α values, a directly recomputed shared aggregate-error threshold, and fixed native-unit physical tolerances. The physical-divergence results reported later in Section 4 favor LSTM rather than Transformer, confirming that the relative score and physical fidelity capture different failure modes. A more conservative operational gate would use shared task-specific native-unit bounds across all models. Such bounds require domain-specific tolerance knowledge; the shared aggregate-error benchmark reported later in Section 4 is a useful common-reference diagnostic but is not a substitute for application-specific physical limits. Our self-scaled criterion remains a benchmark amplification view when such operational tolerances are unavailable.

Plain-language reading. In words, this metric flags a rollout whose largest coordinate error grows to several times the model’s own aggregate 1 s error scale. A model with a larger $\epsilon_{1s}$ receives a wider bound. The metric therefore measures self-scaled amplification on the benchmark coordinate vector, not native-unit physical accuracy. It should be read alongside the shared aggregate-threshold analysis, which applies one common benchmark reference, and the physical-divergence analysis, which applies channel-specific native-unit tolerances. These views can select different models, which is why metric and threshold family must be reported explicitly.

Diagnostic caveat for practical interpretation. This is not a new selector stage, optimization method, or operational gate; it is a reading aid for the self-scaled divergence metric introduced above. A candidate with a substantially larger $\epsilon_{1s}$ is given a correspondingly looser bound, so a favorable self-scaled result should not be read as evidence of favorable physical fidelity. In our benchmark, Transformer’s aggregate RMSE@1s (118.6) is 2.3× GRU’s value (52.3) and nearly 5× LSTM’s value (23.9). Its relative result must therefore be interpreted jointly with the directly measured shared aggregate-threshold and physical-divergence results reported later in Section 4 and with task-specific native-unit tolerances.

### 3.6. Why This Matters for Sensing-Supported Planning

In a practical autonomy stack, rollout quality is judged not only by nominal fit to an underlying trajectory, but also by whether the model remains decision-relevant when its inputs are the current estimated aircraft state. This makes long-horizon behavior relevant for surrogate selection: sensing imperfections and model error can interact across the prediction horizon, so short-horizon RMSE alone is an incomplete benchmark criterion.

### 3.7. Capped RMSE

Standard RMSE hides catastrophic failure because a model with many acceptable rollouts and a few catastrophic ones can still look strong on average. We therefore report capped RMSE, which replaces the RMSE of each diverged rollout with a fixed penalty before computing the mean:

(9) $$\mathrm{Capped}\;{\mathrm{RMSE}}_{20}=\frac{1}{N}\sum_{i=1}^{N}\left\{\begin{array}{cc} C, & \mathrm{if}\;\mathrm{div}.,\\ {\mathrm{RMSE}}_{20}^{\left(i\right)}, & \mathrm{otherwise}, \end{array}\right.$$

where C=500 is the penalty cap, approximately 10× the median RMSE@20s (∼50). This ensures that diverged rollouts receive a substantial penalty without completely dominating the score. Sensitivity analysis shows that varying the cap from 300–700 preserves the top-three ranking (Transformer > GRU > LSTM). Raw RMSE and divergence rate are thus separated into local-fit and amplification views, while capped RMSE recombines them into a single model-selection score that penalizes catastrophic failure more strongly than average error.

### 3.8. Metric Definitions

Three evaluation views: (i) Self-scaled relative divergence marks a rollout as diverged when its largest componentwise simulator-coordinate error exceeds $\alpha \epsilon_{1s}$ (the model’s own aggregate 1 s RMSE scale); (ii) Capped RMSE replaces diverged-rollout aggregate RMSE@20s with penalty C=500 (sensitivity analysis shows the top-three ordering is stable for cap 300–700); and (iii) Shared aggregate-threshold and physical divergence apply one common aggregate benchmark threshold or channel-specific native-unit tolerances, respectively. The aggregate scores combine heterogeneous state coordinates and are therefore diagnostic benchmark quantities; the physical view supplies the interpretable native-unit check. These views can select different models.

### 3.9. Metric-to-Task Correspondence

Each metric family answers a different planning question. Table 5 makes this correspondence explicit so that metric choice is grounded in the actual planning use case rather than treated as interchangeable.

### 3.10. Benchmark Setup and Implementation Details

Five neural surrogate families are compared: MLP (two 128-unit hidden layers, ∼20 K parameters), GRU (two 128-unit recurrent layers, ∼309 K), LSTM (two 128-unit recurrent layers, ∼412 K), TCN (four 128-channel levels, kernel 3, ∼356 K), and Transformer (two encoder layers, $d_{\mathrm{model}}=64$, four heads, ∼69 K). All models use the same 11-dimensional state, four-dimensional control input, context window $T_{c}=50$, and 20 s prediction horizon at 10 Hz (200 steps), but they retain the architecture-native recursive/direct mechanisms described above. State dimensions are standardized to zero mean and unit variance from training data. Main checkpoints use mean-squared-error (MSE) loss and the Adam optimizer (learning rate $10^{-3}$, batch 256, 50 epochs) on JSBSim trajectories from five aircraft: c172p, c182, c310, pa28, and DHC6. Appendix C reports the measured batch-size-1 GPU latency benchmark, under which launch overhead makes all surrogates slower than JSBSim. Because equivalent large-batch sweeps were not collected for all five architectures, we do not make a quantitative cross-model latency claim at batch size 64. Batched candidate-trajectory evaluation remains the intended deployment setting, but its computational advantage requires a controlled architecture-matched benchmark. The central in-distribution benchmark uses 500 windows from the five-aircraft validation split; cross-aircraft evaluation tests transfer to unseen c172r and L410 without fine-tuning. The sensing-aware extension of the benchmark additionally evaluates controlled sensor-interface stress conditions: low/medium/high measurement noise, filtered estimated-state inputs, 100–200 ms delays, dropout, and asynchronous multi-rate refresh, plus a composite condition that combines these effects. These settings are controlled stress-test parameters rather than certified avionics specifications. The abstract-corruption implementation scales each state group by a fraction of its training-set standard deviation; it is not a direct physical sensor-noise model. The separate estimator-loop profiles use native-unit microelectromechanical-system (MEMS) IMU/GNSS/barometric parameters motivated by UAV state-estimation literature [16,17,19]. Table 6 summarises the key parameter ranges used in the abstract sensing-corruption suite.

To move beyond abstract sensing-interface perturbation models, we also add an aircraft-specific estimator-in-the-loop stress test: per-aircraft IMU/GNSS/baro noise is injected into the planner context and passed through a simple complementary/Kalman-style state estimator before surrogate rollout. This fixed estimator path follows the controlled comparative scope defined above; it is not evaluated as a navigation-filter benchmark.

### 3.11. Data Source

All reported numerical results derive from the JSBSim open-source fixed-wing flight-dynamics simulator [1]. The surrogate train/validation/test metadata use five in-distribution aircraft—c172p, c182, c310, pa28, and DHC6—with 80/20/20 trajectories per aircraft; the central open-loop benchmark contains five windows from each of 100 validation trajectories (500 windows per model). Cross-aircraft transfer is evaluated without fine-tuning on held-out c172r and L410 trajectories. Each model is assessed over a 20 s horizon at 10 Hz (200 steps) from a $T_{c}=50$-step (5 s) context through its architecture-native interface. An auxiliary inclusion suite evaluates the rank assigned to the logged control sequence among perturbations for 20 base test trajectories under five sensing conditions; because the perturbed candidates were not re-simulated in JSBSim, this endpoint is not ground-truth candidate-ranking fidelity and is excluded from the task heuristic. The separately generated downstream proxy suite uses c172p, c182, c310, DHC6, and an additional f16 stress aircraft, with two repeats across four task families (40 tracking and 40 screening episodes per scenario and model); its candidate-screening controls are evaluated in JSBSim. The aircraft-specific estimator-loop stress test reports representative c172p, c172r, f16, and L410 settings. These suites share model checkpoints and simulation code but are not the same trajectory split. Existing project metadata, scripts, and result artifacts document provenance; exact regeneration of legacy data is limited by the original process-dependent dataset seeding, which has been corrected in the accompanying generation script for future runs.

### 3.12. Metric Summary

Table 7 consolidates the evaluation metrics used throughout the paper: for each metric it gives the abbreviation, plain-English meaning, the surrogate selected under that criterion on this JSBSim benchmark, the planning task it suits, and its principal limitation. The table is a single reference for both the metric abbreviations and the task-conditioned outcome; the benchmark-specific lexicographic heuristic that combines these metrics is defined in Table 4.

## 4. Results

The Results section is organized into four thematic modules: Section 4.1 establishes the open-loop ranking reversal; Section 4.2 evaluates sensing corruption and cross-aircraft transfer; Section 4.3 examines metric validity and limitations through shared thresholds, metric–task correlation, bootstrap sensitivity, and multi-step training; Section 4.4 assesses physical and downstream task behavior, including estimator-in-the-loop effects. Robustness checks (noise-aware training ablation and five-seed analysis) are in Appendix D.

### 4.1. Short-Horizon Accuracy Reliability When Planning Uses Estimated States

**Figure 2:**
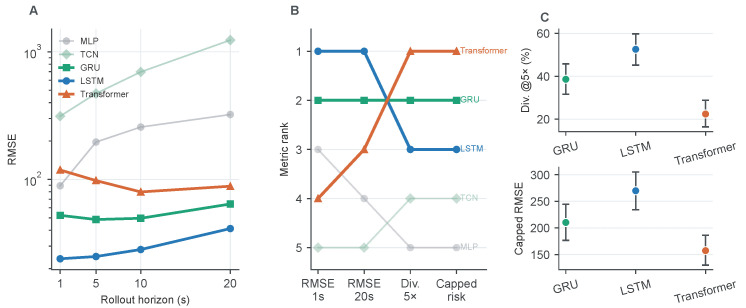
Ranking reversal across rollout-selection metrics. (**A**) RMSE over rollout horizon favors LSTM under local and absolute-error views. (**B**) A bump chart shows that the winner changes when the selection criterion moves from RMSE to self-scaled relative divergence and capped rollout risk. (**C**) Bootstrap intervals for the top three models show that Transformer is preferred under the self-scaled Div@5× and capped-risk criteria, while this does not imply universal absolute-fidelity superiority.

Figure 2 shows the central empirical mismatch: LSTM achieves best aggregate RMSE at both 1 s and 20 s, yet diverges on 52.6% of rollouts under the self-scaled 5× criterion, while Transformer diverges least often (22.4%) and achieves best capped RMSE. This relative ranking flip (Table 8 confirms the numbers) is the main reason short-horizon aggregate RMSE is insufficient for planning-oriented model selection. The main empirical phenomenon is not that Transformer is best, but that no single metric is sufficient; this relative criterion does not imply that Transformer has the smallest absolute long-horizon error.

To avoid over-interpreting the self-scaled threshold, we do not treat Transformer as a universal winner. Instead, we explicitly compare relative, shared-threshold, physical, and downstream criteria. As shown later, the absolute-fidelity views favor LSTM, which is precisely why the proposed protocol outputs a task-conditioned model choice rather than a single best surrogate.

**Figure 3:**
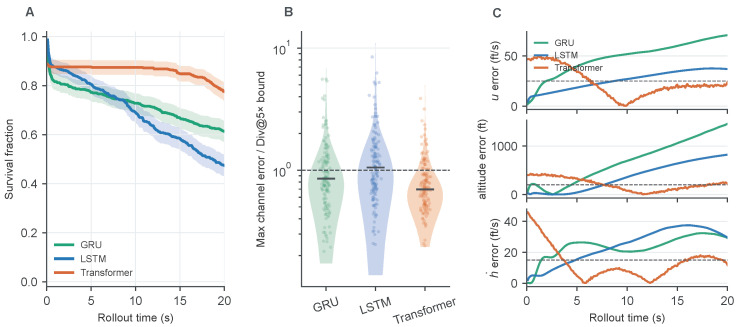
Rollout failure dynamics under architecture-native long-horizon prediction. (**A**) Time-to-divergence survival curves with binomial confidence bands show when each model first crosses the self-scaled Div@5× bound. (**B**) Per-rollout maximum componentwise coordinate-error distributions expose catastrophic-tail behavior rather than only mean error. (**C**) Representative native-unit channel traces show how forward-speed, altitude, and vertical-speed errors are amplified during a 20 s rollout.

Figure 3 summarizes the complementary time-to-divergence, catastrophic-tail, and representative native-unit channel diagnostics.

The broader pattern is also instructive. The feedforward MLP and TCN both exhibit high divergence rates (90.4% and 86.0%), showing that reasonable local fit does not guarantee bounded long-horizon behavior under each architecture’s native rollout interface. Notably, Transformer achieves the lowest divergence rate despite having the highest aggregate RMSE@1s among the top three models (118.6 vs. 23.9 for LSTM and 52.3 for GRU), indicating that short-horizon accuracy and self-scaled error amplification are distinct failure modes: Transformer’s predictions are individually noisier but they trigger fewer failures under the relative-to-own-error criterion, whereas LSTM achieves better local fit and stronger absolute fidelity. GRU occupies an intermediate operating point with lower self-scaled relative amplification than LSTM but better local accuracy than Transformer. Viewed as a selection problem, the benchmark does not ask which architecture is universally best; it asks which model produces task-relevant rollouts inside an autonomous flight planning loop.

### 4.2. Effect of Sensing Delay, Dropout, Filtering, and Multi-Rate Refresh on Rollout Risk

#### 4.2.1. Cross-Aircraft Transfer

The held-out aircraft split is already summarized in Figure 1, so we focus here on the ranking outcome rather than repeating the setup. All three top models degrade substantially on unseen aircraft, confirming that cross-aircraft generalization remains an open challenge. Under this stronger out-of-distribution (OOD) shift, the absolute metric values worsen and some evaluation views become less discriminative, so the results should not be read as evidence of a generally transferable dynamics model. However, the *need* for rollout-aware selection persists under this distribution shift: Transformer still shows the smallest held-out divergence rate in the clean setting (54.3%) and the best held-out capped RMSE (302.1), compared with GRU (77.0%, 392.6) and LSTM (76.3%, 386.7) (Table 9). The accompanying sensing-open-loop artifact also contains held-out evaluations under the abstract corruption operators. Because those conditions exhibit uniformly severe degradation and physical-divergence saturation, Table 9 reports the cleaner and more interpretable clean held-out comparison, while the estimator-loop held-out results are summarized later. The transfer evidence therefore changes the absolute difficulty and reduces confidence in any single winner, but it does not remove the need for task-conditioned surrogate assessment within the stated JSBSim benchmark scenarios.

#### 4.2.2. Sensing Corruption

The central question for a sensing-supported JSBSim benchmark setting is whether the clean-state ranking still matters once planning is driven by measured or estimated states. To test this, we evaluate five sensing-aware context conditions: medium measurement noise, filtered estimated-state input, 200 ms delay, dropout and multirate refresh, and a composite condition combining medium noise, filtering, delay, dropout, and asynchronous sensing.

**Figure 4:**
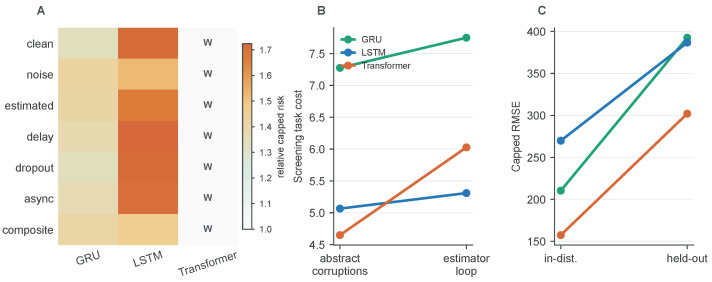
Sensing, estimator-path, and transfer robustness. (**A**) Scenario–model atlas of normalized capped rollout risk under in-distribution sensing conditions; the marked cell indicates the winner within each scenario. (**B**) Abstract sensing corruptions favor Transformer for trajectory screening, whereas the aircraft-specific estimator loop shifts screening cost toward LSTM. (**C**) Held-out aircraft substantially increase capped risk for all models, but the metric-dependent ranking disagreement remains visible.

Table 10 shows that the ranking reversal is not a clean-state artifact. Across all sensing-aware variants, LSTM remains the RMSE@20s winner, whereas Transformer remains the divergence and capped-RMSE winner. The filtered estimated-state condition stays close to clean evaluation, while medium measurement noise and the composite condition slightly increase RMSE for both models. The ranking itself, however, is unchanged. This is the key practical result for sensing-supported planning: the metric disagreement survives when the planner consumes simplified measured or estimated states. Figure 4 visualizes this robustness pattern and also highlights the estimator-loop screening flip.

The absolute-fidelity view also remains informative. Under all in-distribution sensing conditions, LSTM continues to have lower physical-divergence rate than Transformer, while on held-out aircraft the physical-divergence metric saturates near 100% for all models and becomes less discriminative. Per-state analysis shows that the dominant sensing-aware failure drivers in-distribution remain heading, vertical-speed, and altitude channels, while the held-out setting additionally exposes forward-speed and roll-angle degradation. In other words, the ranking flip is still driven by long-horizon pose and energy-state consistency rather than by instantaneous rate prediction.

### 4.3. Metric–Task Alignment for Downstream Selection

#### 4.3.1. Shared Thresholds and Metric–Task Correlation

The main divergence metric uses a per-model threshold, so we also directly recompute failures under a shared aggregate-error threshold defined as 5× the cross-model median validation RMSE@1s in the same denormalized simulator-coordinate vector. Across all five model families, the median reference is 89.14, giving a common threshold of 445.69 in this heterogeneous-coordinate benchmark scale. Across 500 validation rollout windows per model, the ranking changes (Table 11): LSTM becomes the least-failing model (6.6%), followed by GRU (12.8%) and Transformer (43.0%). This means the strongest version of our claim should not be that self-scaled relative divergence alone universally identifies the best benchmark model. Instead, the defensible conclusion is narrower: different rollout-evaluation views select different models, and self-scaled relative divergence should be interpreted together with capped RMSE, physical divergence, and directly simulated downstream task results.

We make that point explicit by correlating open-loop metrics with downstream behavior across 18 model/scenario points (GRU, LSTM, and Transformer over five abstract sensing conditions plus the aircraft-specific estimator loop). Table 12 shows that divergence and capped RMSE have the strongest positive descriptive association with screening cost (0.398 and 0.402), whereas tracking cost is only weakly correlated with any single open-loop metric. The 18 points share models, sensing conditions, and source simulations, so these coefficients are descriptive associations rather than independent-sample inference. The more precise claim supported by the data is therefore that relative-amplification metrics align more closely with the evaluated screening cost than short-horizon aggregate RMSE does, while no single metric is sufficient for all downstream tasks.

#### 4.3.2. Bootstrap Confidence Intervals and Threshold Sensitivity

The 95% intervals use a trajectory-cluster bootstrap over the 100 source validation trajectories, retaining all five evaluation windows from each sampled trajectory, and do not require retraining. They preserve the top-three ordering under the main self-scaled 5× criterion: Transformer remains preferred under both relative divergence rate and capped RMSE, followed by GRU and then LSTM. This ordering is unchanged when the divergence multiplier is tightened or loosened to 3× or 7×, which mitigates the concern that the ranking is an artifact of a single arbitrary threshold. The sensing-aware sweeps lead to the same qualitative conclusion: noise, filtering, delay, dropout, and asynchronous refresh change the metric values, but not the fact that aggregate RMSE and self-scaled relative-amplification-oriented criteria reward different models.

Table 13 reports the trajectory-cluster bootstrap intervals and threshold-sensitivity results.

#### 4.3.3. Multi-Step Training Can Improve Local Error While Harming Rollout Usability

The GRU ablation shows that better local accuracy does not automatically translate into better long-horizon behavior. Training with multi-step loss reduces GRU RMSE@1s from 52.3 to 10.9, yet the divergence rate rises from 1.8% to 28.0% (under the ablation’s per-model threshold). This makes the model look better under conventional error metrics while making it less suitable for architecture-native rollout stress. For future planning-oriented evaluation, multi-step objectives should therefore be assessed jointly with divergence-aware criteria rather than assumed to be an unconditional improvement.

### 4.4. Effect of the Estimator Path on Surrogate Selection

The main divergence metric uses a per-model relative threshold ($\alpha \epsilon_{1s}$), which emphasizes error amplification relative to each model’s own short-horizon scale. A complementary engineering question is whether rollout error violates fixed physical tolerances in native units. We therefore add a second view in which a rollout is marked as failed if any state dimension exceeds its tolerance at any step. The thresholds are selected as strict engineering stress tolerances for light-to-medium aircraft: 25 ft/s forward-speed error, 10° attitude error, 15° heading error, 200 ft altitude error, and 15 ft/s vertical-speed error, with analogous tolerances for the remaining states. These values are engineering-estimate tolerances rather than certification-derived limits, not intended as safety certification thresholds, provided as a task-agnostic sanity check on absolute-error scale alongside the main self-scaled relative-amplification metric. Sensitivity analysis from 0.5× to 1.5× the reported tolerances consistently leaves LSTM as the least-failing top model. At the strictest 0.5× setting, GRU and Transformer both reach 100% failure, so their mutual ordering is tied rather than strictly preserved.

Table 14 shows that this absolute-error view reorders the top models. Under the main self-scaled relative criterion, Transformer remains preferred (22.4%), followed by GRU (38.6%) and LSTM (52.6%). Under the fixed physical-tolerance criterion, however, LSTM becomes the least-failing top model (90.4%), followed by GRU (98.2%), while Transformer fails on all rollouts because its absolute forward-speed, altitude, and vertical-speed errors are larger despite lower self-scaled relative amplification. This distinction remains visible under the sensing-aware in-distribution variants as well: Transformer remains preferred by self-scaled relative amplification, whereas LSTM remains strongest by absolute fidelity. These physical-divergence rates should be interpreted as comparative stress indicators rather than evidence of immediate field readiness. The high physical-divergence rates indicate that the evaluated surrogates should be interpreted as selection candidates under rollout stress rather than operational replacements for high-fidelity flight dynamics; under these strict native-unit tolerances, the protocol rejects all candidates for state-faithful operational use. The purpose of the selection protocol is therefore not to certify any surrogate as physically reliable under strict native-unit tolerances, but to expose how different evaluation criteria identify different failure modes before planner integration. This result strengthens the motivation for sensing-aware evaluation: conventional RMSE-based model choice would hide these physical failure modes, making the proposed multi-view protocol necessary rather than optional.

An apparent tension arises between this result and the selector table (Table 4), which recommends Transformer for candidate screening under abstract sensing corruption. This is not a contradiction: the lexicographic selector for screening prioritizes the directly simulated screening cost $m_{\mathrm{task}}$ and bounded rollout risk $m_{\mathrm{cap}}$ (capped RMSE with penalty C=500). The physical divergence metric $m_{\mathrm{abs}}$ uses strict native-unit stress tolerances under which all three top models fail at high rates (90–100%), making it a poor discriminator for screening tasks where relative ordering matters more than absolute error scale. The reported benchmark heuristic places $m_{\mathrm{abs}}$ below the observed screening cost and capped-risk metrics for abstract screening and raises physical or energy-state fidelity for state-faithful prediction, feedback-rich tracking, and estimator-loop assessment.

**Figure 5:**
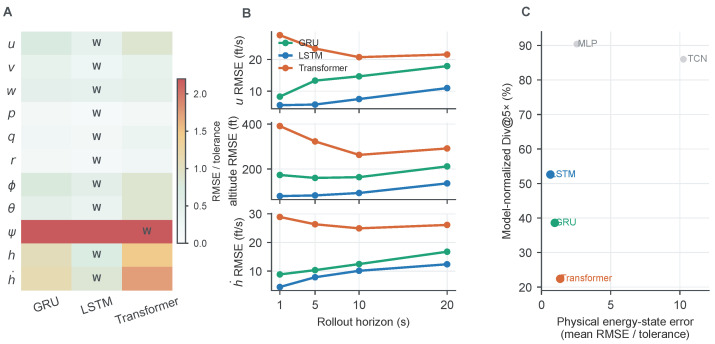
Physical absolute-error fidelity and relative-amplification trade-off. (**A**) Channel-level native-unit RMSE normalized by strict engineering stress tolerances shows that LSTM provides the strongest absolute-fidelity profile. (**B**) Energy-state RMSE curves show lower forward-speed, altitude, and vertical-speed error for LSTM across the rollout horizon. (**C**) A Pareto-style view separates physical energy-state fidelity from self-scaled relative divergence, illustrating why the protocol should output task-conditioned choices rather than a universal surrogate winner.

Figure 5 visualizes the channel-level source of this trade-off.

#### 4.4.1. Auxiliary Logged-Control Inclusion Diagnostic

For transparency, we retain an auxiliary diagnostic that places the logged control sequence among 49 perturbed candidates scored by each surrogate, repeated over 20 source trajectories and the sensing-aware context conditions. Only the logged control sequence has a corresponding JSBSim state trajectory; the perturbed candidates were not re-simulated in JSBSim in this diagnostic. Consequently, the reported position and top-5 inclusion rate measure where a surrogate places the logged sequence within its own predicted candidate set; they do not measure ground-truth candidate ranking fidelity.

Table 15 shows a large difference in this logged-sequence inclusion heuristic, but the absence of JSBSim outcomes for the perturbed candidates prevents a fidelity interpretation. We therefore exclude this diagnostic from the selector definition, metric–task correlation analysis, headline findings, and inferential claims; the directly simulated 64-candidate screening suite below provides the valid candidate-selection evidence.

#### 4.4.2. Aircraft-Specific Estimator-in-the-Loop Evaluation

The previous sensing suite still uses abstract corruption operators applied directly to the planner context. To address the more concrete sensing concern, we add an aircraft-specific estimator-in-the-loop stress test in which each aircraft is assigned its own IMU/GNSS/baro noise profile and update rates. The planner does not receive corrupted ground-truth states directly. Instead, noisy gyro, velocity, altitude, and vertical-speed channels are passed through a simple complementary/Kalman-style estimator: attitude is propagated from gyro measurements and corrected by low-rate attitude observations, while body velocity and altitude channels are updated by scalar innovation steps driven by GNSS and barometric measurements. All candidates use the same fixed estimator path defined in Section 3; this experiment compares surrogate behavior under that path rather than navigation-filter performance, while remaining materially closer to an onboard sensing-estimation-planning chain than the earlier benchmark-side corruption operators.

Table 16 shows three important points. First, the main open-loop disagreement survives this estimated-state interface: LSTM still has the best RMSE@20s (55.6), while Transformer still has the best divergence rate (24.4%) and capped RMSE (167.0). The same held-out pattern also persists under this estimator loop, where Transformer remains preferred under held-out divergence and capped RMSE (52.7%, 295.2), ahead of GRU (77.0%, 392.5) and LSTM (80.0%, 404.8). Second, the auxiliary logged-control inclusion diagnostic places the logged sequence higher under Transformer, but, as above, it is not a ground-truth ranking endpoint and is not used by the selector. Third, the directly simulated downstream story becomes more nuanced. Closed-loop tracking still slightly favors Transformer by task cost (2.953 vs. 3.197), although LSTM retains slightly higher success (92.5% vs. 90.0%). Trajectory screening, however, now flips toward LSTM under this simple estimator loop: LSTM achieves lower screening task cost (5.309 vs. 6.027) and much higher top-5 hit rate (37.5% vs. 5.0%). This selector-path flip should be interpreted as estimator-path dependence rather than inconsistency. The aircraft-specific estimator loop and the abstract sensing-corruption suite induce different planner-facing state distributions and different screening-cost sensitivities, which reinforces the need for estimator-conditioned model selection. The channel-level comparison suggests why this happens. Under the estimator loop, Transformer still has lower self-scaled relative amplification, but LSTM reduces the largest absolute errors in altitude, vertical speed, and forward speed: its altitude RMSE@20s is 183.2 ft versus 297.5 ft for Transformer, its vertical-speed error is 16.8 ft/s versus 26.6 ft/s, and its forward-speed error is 13.6 ft/s versus 22.3 ft/s. Transformer is only slightly better on heading (1.55 rad versus 1.63 rad). The normalized energy-state metric $m_{\mathrm{energy}}=\sqrt{\sum_{j}{\left({\mathrm{RMSE}}_{20,j}/\sigma_{j}\right)}^{2}}$ over forward speed, altitude, and vertical speed is 0.557 for LSTM versus 0.895 for Transformer under the estimator loop (see Table 16), confirming that LSTM’s absolute energy-state advantage is consistent across the individual channels and drives the estimator-loop selector to prioritize $m_{\mathrm{energy}}$ first. Under the abstract sensing-corruption suite, $m_{\mathrm{energy}}$ on average remains lower for LSTM than for Transformer, consistent with the in-distribution per-channel altitude, vertical-speed, and forward-speed components reported in Table 10. Even so, the selector for abstract-corruption screening prioritizes the directly measured screening cost $m_{\mathrm{task}}$, for which Transformer is lower in all five reported conditions. This pattern is consistent with the downstream flip: the estimator smooths high-frequency attitude noise and changes the planner-facing state distribution, while LSTM’s stronger absolute energy-state fidelity becomes more important for the screening cost actually used in the downstream benchmark.

#### 4.4.3. Exploratory Downstream Check (Screening and Tracking)

The original small MPC sanity check is replaced here by a larger sensing-aware downstream suite: 40 closed-loop tracking episodes and 40 candidate-screening episodes per scenario and per model, covering five aircraft, four task families, and disturbed execution. The full suite evaluates GRU, LSTM, and Transformer for metric-correlation and selector analysis, while the main table focuses on LSTM and Transformer as the two models representing the dominant absolute-fidelity–relative-amplification trade-off.

**Figure 6:**
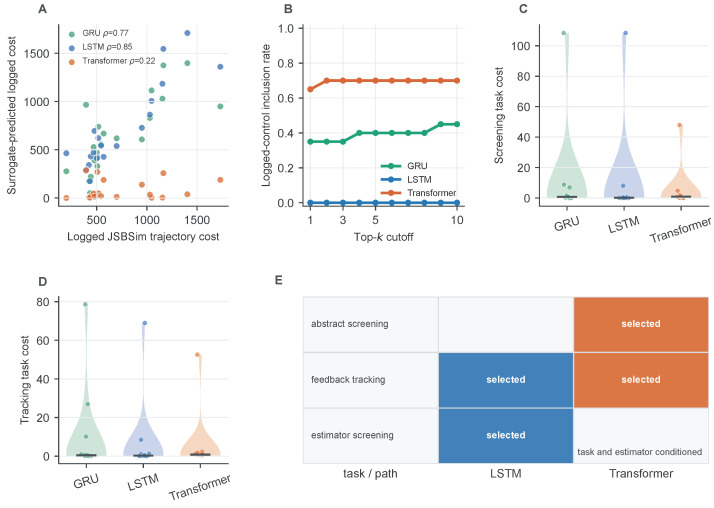
Downstream task assessment of metric-dependent surrogate selection. (**A**) Nominal logged-trajectory score and (**B**) logged-control top-*k* inclusion summarize the auxiliary candidate-set diagnostic; because perturbed candidates lack JSBSim ground truth, these panels are not ranking-fidelity evidence. (**C**,**D**) Screening and feedback-tracking task-cost distributions expose different sensitivity to rollout errors and catastrophic episodes. (**E**) The resulting benchmark heuristic is conditioned on both task and estimator path: directly simulated screening cost favors Transformer under abstract corruption, while estimator-loop screening can shift toward LSTM because physical channel fidelity becomes more important.

Table 17 shows that sensing corruption narrows but does not erase task dependence. For trajectory screening, Transformer has the lower observed cost in every evaluated abstract-corruption scenario, which is consistent with the open-loop relative-amplification results. For closed-loop tracking, the gap is smaller: Transformer yields lower tracking task cost in clean, noisy, estimated, and delayed settings, whereas LSTM is marginally better in the composite condition. Success rates are also close rather than decisive: under the clean setting, tracking success is 90.0% for Transformer and 92.5% for LSTM (Table 17). With 40 episodes, Wilson 95% intervals are wide enough that 2.5–5.0 percentage-point success-rate gaps should not be interpreted as statistically decisive. This is the benchmark-level conclusion we wanted to test: metric choice changes model choice, and different sensing-supported planning tasks can still prefer different models even after we move beyond clean open-loop evaluation. Figure 6 summarizes the corresponding auxiliary inclusion, directly simulated screening, tracking, and selector-level behavior.

The decision-rule comparison reinforces this point. Across the abstract sensing-aware scenarios, RMSE@20s continues to select LSTM, while self-scaled divergence and capped RMSE continue to select Transformer. The downstream suite then shows the consequence of that choice: the RMSE-selected model remains competitive in feedback-rich tracking, whereas the self-scaled relative-amplification-selected model remains consistently better for candidate screening.

#### 4.4.4. Assessment of the Task- and Estimator-Conditioned Screening Protocol

We also compare the transparent operator in Table 4 with the reported downstream proxies. This comparison assesses descriptive consistency of the screening heuristic; it is not an independent validation stage. Using validation-set rollout metrics together with the directly simulated downstream summaries, we compare five decision rules: RMSE@20s, self-scaled relative divergence, capped RMSE, physical-fidelity, and the reported task-conditioned benchmark heuristic. The first four choose a single global model, whereas the task-conditioned heuristic explicitly allows different choices for different downstream uses and estimator paths. In the abstract sensing-corruption suite, screening follows the observed task-cost and self-scaled relative-amplification evidence. Tracking endpoints are mixed: Transformer has slightly lower mean cost, whereas LSTM has slightly higher success. In the aircraft-specific estimator loop, however, screening can shift toward LSTM because the planner-facing state distribution makes absolute energy-state fidelity more important.

Table 18 summarizes descriptive consistency between the reported metric heuristics and downstream proxy outcomes; it is not an independently held-out selector-validation table. Relative-amplification-based selectors choose Transformer and then obtain lower mean screening cost (4.650 vs. 5.058 for LSTM-based selectors, approximately 8% difference). Fidelity-based selectors choose LSTM and then preserve the stronger tracking-success profile (92.5% vs. 90.5%), even though the mean tracking cost itself is slightly lower for Transformer. Given the wide Wilson 95% intervals at 40 episodes, the 2.0-percentage-point tracking-success gap should be interpreted descriptively rather than as statistically decisive; the approximately 8% screening-cost gap is also descriptive. Most importantly, the reported heuristic makes these two levels of conditioning explicit: task type determines which metric family is prioritized, and the estimator path determines whether downstream task cost or physical channel fidelity dominates within the same task. This is the core protocol-level result: the sensing-aware rollout evaluation provides a transparent task- and estimator-conditioned benchmark heuristic with an explicit reported priority order. For candidate screening, the directly simulated evidence shows a modest descriptive cost difference across the evaluated abstract-corruption scenarios. For feedback-rich tracking, mean cost and success favor different models and the evidence remains preliminary at n=40 per scenario. The protocol therefore provides benchmark-specific, hypothesis-generating guidance rather than an independently validated operational selector.

Robustness checks (noise-aware training ablation and five-seed winner-frequency analysis) are reported in Appendix D. In brief: LSTM wins RMSE@20s in 5/5 seeds and Transformer wins self-scaled Div@5× and capped RMSE in 5/5 seeds, confirming that the in-distribution ranking disagreement is stable across training runs; held-out transfer rankings are less stable and should be interpreted with explicit uncertainty.

## 5. Discussion: Practical Guidance and Scope of Validity

### 5.1. Match Selection Criterion and Horizon to Planning Task

Different planning-oriented metrics can select different models: batched trajectory evaluation can prioritize low self-scaled amplification; state-faithful prediction prioritizes native-unit fidelity; directly simulated candidate screening prioritizes task cost; and feedback-rich tracking requires joint inspection of cost and success because the two endpoints favor different models in this study. The evaluation horizon should reflect the intended planning window; 20 s in our setting exposes metric-ordering reversals invisible at short horizons. For sensing-supported autonomous flight, this matching step should be done with the onboard state pipeline in mind, because the planner consumes measured or estimated states rather than simulator truth.

### 5.2. Treat Multi-Step Training as a Tradeoff

The GRU ablation shows rollout-aware training can improve accuracy metrics while worsening catastrophic failure rate. Training objectives should be judged by the same rollout-usability criteria used for final model selection. The noise-aware training comparison extends this warning: naively injecting noisy context states during training worsens sensing-corrupted test performance for both LSTM and Transformer, so sensing robustness is not obtained for free by simple corruption augmentation.

### 5.3. Use the Protocol as a Task-Conditioned Screening Heuristic

Different criteria favor different architectures on this benchmark: short-horizon aggregate RMSE, directly measured shared aggregate-threshold failure, and native-unit absolute fidelity favor LSTM; self-scaled relative-amplification and capped-RMSE criteria favor Transformer; and directly simulated screening cost favors Transformer under the abstract corruption suite but favors LSTM under the aircraft-specific estimator loop. It is important to note that these metric families answer different questions and should not be collapsed into a single universal ranking: absolute physical thresholds measure native-unit fidelity, self-scaled amplification measures relative growth on a heterogeneous simulator-coordinate benchmark score, and downstream task metrics measure planning-relevant behavior. The practical output of the proposed protocol is therefore a task-conditioned heuristic rather than a single winner. The transparent benchmark-specific priority order is defined in Table 4; Table 19 translates that order into task-conditioned guidance for the main cases described by the experiments. This is especially important for sensing-supported autonomous systems, where the planning stack depends on the interaction between onboard sensing, state estimation, and long-horizon model rollout. The aircraft-specific estimator-in-the-loop experiment sharpens this point further: Transformer remains lower under self-scaled open-loop divergence under the estimated-state interface, yet downstream trajectory screening flips toward LSTM once the planner consumes a concrete sensor-driven state estimate. Model selection should therefore be assessed against the actual estimator path seen by the planner, not only against abstract benchmark-side noise injection. The directly recomputed shared aggregate-threshold analysis makes the same point from another angle: once all models are evaluated under one common aggregate benchmark threshold, LSTM rather than Transformer becomes the least-failing model. Self-scaled relative divergence is therefore useful, but it should be treated as one diagnostic view rather than as a universal surrogate-selection oracle.

### 5.4. Estimator Fidelity and Expected Selection Sensitivity

The estimator-in-the-loop experiment uses a lightweight complementary/Kalman-style estimator to isolate how planner-facing state uncertainty changes surrogate rollout. An industrial EKF/UKF with online bias estimation, cross-channel covariance updates, and explicit air-data/IMU/GNSS coupling would likely reshape the estimated-state error spectrum. Bias-state estimation and covariance-consistent fusion would reduce low-frequency drift and cross-axis inconsistency, which should help recurrent models such as LSTM when their absolute energy-state errors are driven by slowly varying altitude, vertical-speed, or forward-speed offsets. At the same time, stronger filtering and multi-rate smoothing could attenuate high-frequency attitude and rate perturbations, potentially changing the relative-amplification advantage that Transformer shows in some rollouts. The expected outcome is not a universal flip, but a narrower and estimator-dependent tradeoff: LSTM should remain competitive or stronger when absolute state fidelity dominates, whereas Transformer may remain attractive when downstream screening cost is more sensitive to relative amplification than to absolute offsets. This is why the protocol treats the estimator path as part of the evaluation condition rather than assuming a fixed architecture ranking.

### 5.5. Scope of Validity

The conclusions should be read as evidence about sensor-interface-sensitive evaluation within controlled JSBSim fixed-wing benchmarks. They do not establish field performance, real-sensor robustness, or cross-platform transfer without additional hardware, estimator, and flight-test validation. We state the scope boundary explicitly: the core train/validation/test metadata cover five in-distribution JSBSim aircraft (c172p, c182, c310, pa28, DHC6) and two held-out aircraft (c172r, L410). The separately generated downstream proxy suite uses c172p, c182, c310, DHC6, and an additional f16 stress aircraft, while the aircraft-specific estimator-loop table reports representative c172p/c172r/f16/L410 settings. This coverage is deliberately structured so that the sensor-to-planner interface can be varied under controlled environments; the suites are related through common checkpoints and simulation code but do not share one identical trajectory split. Accordingly, JSBSim-only coverage does *not* establish cross-simulator, real-flight, or broad aircraft-class generality, and the reported rankings should not be extrapolated beyond the stated suite-specific aircraft sets. Broader cross-aircraft and cross-simulator coverage, and validation against higher-fidelity estimators, are the primary lines of future work.

### 5.6. Limitations

The five-seed winner-frequency analysis covers all main architectures, but statistical power remains limited and several pairwise tests stay above conventional p<0.05 thresholds because only five seeds are available. The self-scaled relative divergence criterion is benchmark-oriented, combines heterogeneous simulator-coordinate units, and is not a full downstream task metric. The reported metric views are compared with outcomes from disturbed MPC tracking and candidate-screening loops, but the task-conditioned heuristic is not independently validated inside a full policy-improvement, online replanning, or learning-to-control pipeline. Cross-aircraft transfer remains limited, which reinforces that rollout-aware model selection is a necessary criterion for future operational evaluation, not a complete solution to generalization. Finally, the divergence metric’s relative threshold design is intentionally self-scaled and may favor models with higher $\epsilon_{1s}$; we contextualize this through the multiplier sweep, the shared aggregate-threshold diagnostic, and fixed native-unit physical checks; task-specific physical tolerances would provide stronger guarantees when domain knowledge is available. These absolute-error results should be interpreted as comparative operational stress indicators rather than evidence of immediate field readiness. These findings remain conditioned on the fixed lightweight estimator path defined in Section 3 and do not validate a navigation filter; higher-fidelity EKF/UKF and hardware-in-the-loop evaluation remain future work.

## 6. Conclusions

This study yields three concrete takeaways. (1) Metric and threshold family change the benchmark ordering: self-scaled relative-amplification criteria favor Transformer, whereas the directly measured shared aggregate threshold favors LSTM (6.6% failure, versus 12.8% for GRU and 43.0% for Transformer) and native-unit physical-fidelity criteria also favor LSTM. The aggregate RMSE/divergence scores combine heterogeneous simulator-coordinate units and are benchmark diagnostics rather than physical safety margins. The self-scaled ordering is stable across α∈{3,5,7} and the capped-RMSE ordering across C∈[300,700]. (2) Task and estimator path change the relevant evidence: directly simulated candidate-screening cost is approximately 8% lower for Transformer across the five abstract corruption conditions, but the aircraft-specific estimator loop reverses that observed screening-cost ordering in favor of LSTM. Tracking remains exploratory at 40 episodes per scenario and its cost and success endpoints are mixed. These results support a transparent benchmark heuristic, not an independently validated operational selector. (3) No surrogate is field-ready: under strict native-unit tolerances the top models fail on 90–100% of rollouts, so all results are comparative stress-test evidence rather than operational-readiness evidence.

This paper presents a JSBSim sensor-interface evaluation protocol for task-conditioned assessment of learned fixed-wing flight-dynamics surrogates. The core surrogate splits use c172p/c182/c310/pa28/DHC6 in distribution and c172r/L410 held out; separately generated downstream and estimator-loop suites use the explicitly reported suite-specific aircraft sets. The aircraft-specific estimator-in-the-loop and metric–task correlation findings detailed in Section 4 and Section 5 reinforce the same conclusion: changing the sensing-estimation interface can change which surrogate and evaluation metric appear preferable, so clean-state accuracy alone is insufficient. We do not claim these rankings generalize beyond the tested JSBSim aircraft; cross-aircraft transfer shows substantial degradation and reduced metric discriminability under severe distribution shift and is treated as a limitation (see below), not as evidence of universal transferability. The contribution is therefore a simulation-based pre-operational evaluation and task-conditioned selection protocol, not a learned dynamics model with strong cross-aircraft generalization.

### Limitations and Scope

This study evaluates sensor-interface-conditioned surrogate selection under a fixed lightweight estimator abstraction, not certified flight-state estimation, navigation, or control. The sensor settings are controlled stress conditions and the downstream checks are proxy-level; they do not constitute flight certification. Although five-seed robustness analysis is included for all main architectures, statistical power remains limited and broader multi-run validation would still be valuable. The trajectory-cluster bootstrap captures validation-trajectory sampling uncertainty only and does not capture training stochasticity. Cross-aircraft transfer is limited, confirming that rollout-aware selection is necessary but insufficient under severe OOD shift. These results do not establish cross-aircraft generalization of rollout-usability criteria beyond the stated JSBSim suite-specific aircraft sets; transfer findings are indicative, not definitive evidence that the proposed evaluation heuristic transfers universally. The retained logged-control inclusion diagnostic is not a ground-truth ranking measure because the perturbed candidates were not re-simulated in JSBSim; it is excluded from the selector and headline claims. The larger model-predictive-control tracking and candidate-screening suite provides the directly simulated downstream evidence. All conclusions should therefore be read as applying within the tested JSBSim setting and sensor/estimator abstractions, while future work will integrate full EKF/UKF navigation stacks and hardware-in-the-loop validation.

Next sensing-aware step. The next step is to integrate a full aircraft-aware navigation stack and validate the resulting planner interface in hardware-in-the-loop or flight experiments.

## Figures and Tables

**Table 1:** Sensor-interface contribution map. Each row links a sensing/estimation element to its uncertainty mechanism, planner-facing state effect, and the evaluation metric that captures it. The five surrogate architectures are the empirical vehicle, not the primary contribution. Abbreviations: IMU, inertial measurement unit; GNSS, global navigation satellite system; RMSE, root-mean-square error; Div, divergence.

Sensing Element	Uncertainty Mechanism	Planner-State Effect	Evaluation Metric
IMU attitude/rate	Gyro noise, bias drift	Attitude/rate error in context window	RMSE@1s, divergence rate
GNSS/air-data velocity	Measurement noise, multi-rate refresh	Velocity channel uncertainty	RMSE@20s, capped RMSE
Barometric altitude	Noise, slow drift	Altitude offset in rollout input	Physical divergence (*h* channel)
Sensing delay (100–200 ms)	Stale context states	Increased rollout error amplification	Divergence at the 5× threshold (Div@5×)
Dropout/async refresh	Missing or asynchronous channels	Sparse/inconsistent context	Capped RMSE, screening cost
Estimator filtering	Complementary/Kalman smoothing	Smoothed but lagged state estimates	Estimator-loop selector flip

**Table 2:** Aircraft-specific sensor and estimator settings for the estimator-in-the-loop experiment. The rate/use column reports the nominal update rate and how each sensed channel enters the lightweight planner-facing estimator. Exact per-aircraft parameters are given in Appendix A.

Source	Channels	Noise std	Bias/Drift	Update Rate/Planner-Estimator Use
IMU gyro	p,q,r	0.010–0.020 rad/s	0.0010–0.0022 rad/s RW	10 Hz, attitude propagation
Attitude obs.	ϕ,θ,ψ	0.8–1.4°, 1.4–2.5° heading	none	10 Hz, complementary correction
GNSS velocity	u,v,w	1.2–3.0 ft/s	0.08–0.16 ft/s RW	5–10 Hz, velocity update
Barometer	*h*	8–16 ft	0.35–0.80 ft RW	5–10 Hz, altitude update
Vertical speed	$\dot{h}$	1.0–2.2 ft/s	0.05–0.09 ft/s RW	5–10 Hz, climb-rate update

**Table 3:** Explicit metric definitions used in the task-conditioned benchmark heuristic. Metrics are drawn from the reported open-loop and directly simulated downstream suites; lower is better unless noted.

Metric	Scalar Formula	Aggregation	Direction
$m_{\mathrm{local}}$	RMSE@1s (Equation (7))	Mean over rollouts	Lower better
$m_{\mathrm{rel}}$	Div. rate under $\alpha \epsilon_{1s}$ (Equation (8))	Fraction	Lower better
$m_{\mathrm{cap}}$	Capped RMSE@20s (Equation (9))	Mean with penalty C=500	Lower better
$m_{\mathrm{abs}}$	Physical-div. rate (tolerances: u<25 ft/s, h<200 ft, $\dot{h}<15$ ft/s)	Fraction	Lower better
$m_{\mathrm{task}}$	Screening/tracking cost	Mean over episodes	Lower better
$m_{\mathrm{energy}}$	$l_{2}$ norm of channel RMSE@20s divided by training σ	Global channel-RMSE composite	Lower better

**Table 4:** Explicit definition of the transparent task- and estimator-conditioned benchmark heuristic used in this paper. Each row reports the metric tuple minimized lexicographically for a target planning task and estimator path; the tuples summarize the observed metric–task relationships and are not presented as preregistered or independently held-out selector validation.

Use Case τ	Estimator Path *e*	Lexicographic Selector $\Phi_{\tau ,e}$
Candidate screening	Abstract sensing corruption	$\left(m_{\mathrm{task}},\;m_{\mathrm{cap}},\;m_{\mathrm{rel}},\;m_{\mathrm{abs}},\;m_{\mathrm{local}}\right)$
Candidate screening	Aircraft-specific estimator loop	$\left(m_{\mathrm{task}},\;m_{\mathrm{energy}},\;m_{\mathrm{cap}},\;m_{\mathrm{rel}},\;m_{\mathrm{abs}}\right)$
State-faithful prediction	Any path	$\left(m_{\mathrm{abs}},\;m_{\mathrm{energy}},\;m_{\mathrm{local}},\;m_{\mathrm{rel}}\right)$
Feedback-rich tracking	Any path	$\left(m_{\mathrm{abs}},\;m_{\mathrm{task}},\;m_{\mathrm{energy}},\;m_{\mathrm{local}}\right)$
Cross-aircraft transfer assessment	Held-out aircraft	$\left(m_{\mathrm{abs}},\;m_{\mathrm{cap}},\;m_{\mathrm{rel}},\;m_{\mathrm{local}}\right)$

**Table 5:** Metric-to-task correspondence. Each row maps a rollout metric to the planning question it answers and the use case where it is the primary selection criterion.

Metric	Planning Question Answered	Primary Use Case
Short-horizon RMSE ($m_{\mathrm{local}}$)	Which model fits local dynamics most accurately?	Baseline comparison; not sufficient alone for planning
Self-scaled relative divergence ($m_{\mathrm{rel}}$)	Does the largest coordinate error amplify relative to the model’s own aggregate short-horizon scale?	Candidate screening/long-horizon stress assessment
Capped RMSE ($m_{\mathrm{cap}}$)	Does the model avoid catastrophic rollout failure?	Candidate screening with tail-risk penalty
Physical and energy-state fidelity ($m_{\mathrm{abs}}$, $m_{\mathrm{energy}}$)	Does the model retain absolute channel fidelity after accounting for channel scale?	State-faithful prediction; feedback-rich tracking
Logged-control inclusion proxy ($m_{\mathrm{incl}}$)	Where does each surrogate rank the recorded control sequence among random perturbations?	Auxiliary diagnostic only; perturbed candidates lack JSBSim ground-truth costs
Downstream task outcome ($m_{\mathrm{task}}$)	What cost or success is observed in the directly simulated screening or tracking task?	Exploratory downstream assessment

**Table 6:** Implemented stress-test parameters for the abstract sensing-corruption suite. Noise is applied as a fraction of each channel’s training-set standard deviation; it is not a direct physical sensor specification. Per-aircraft native-unit estimator-loop parameters are in Appendix A.

State Group	Noise Scale × Training σ	Delay/Refresh	Implementation Note
Velocity channels (u,v,w)	Low 0.015; medium 0.035; high 0.070	Optional asynchronous refresh	Applied to the stored training standard deviation of each velocity channel
Attitude and rate channels	Low 0.012; medium 0.028; high 0.055	Optional estimator filtering	Applied separately to p,q,r,ϕ,θ,ψ training scales
Altitude and vertical speed	Low 0.020; medium 0.050; high 0.100	Optional estimator filtering	Applied separately to *h* and $\dot{h}$ training scales
Sensing delay	—	100 ms/200 ms	Context is shifted by one or two 10 Hz samples
Dropout/asynchronous refresh	5%/10% dropout	Multi-rate 5–20 Hz	Controlled benchmark corruption

**Table 7:** Metric summary: for each rollout-evaluation metric, its abbreviation, meaning, the surrogate it selects under the reported criterion on this JSBSim fixed-wing benchmark, the suitable planning task, and its principal limitation. Model selections are benchmark-specific outcomes, not universal rankings; the physical-divergence results reported later show that all candidates fail under strict native-unit tolerances.

Metric (Abbrev.)	Meaning	Selected Model	Suitable Use/Limitation
RMSE@1s ($m_{\mathrm{local}}$)	Aggregate short-horizon prediction error	LSTM	Local accuracy; heterogeneous-unit benchmark score, not physical error
Self-scaled divergence ($m_{\mathrm{rel}}$)	Largest coordinate error vs. each model’s own aggregate 1 s scale	Transformer	Long-horizon stress screening; heterogeneous-unit benchmark score
Capped RMSE ($m_{\mathrm{cap}}$)	Aggregate RMSE@20s with diverged rollouts penalized (C=500)	Transformer	Tail-risk screening; cap- and coordinate-scale-dependent
Physical divergence ($m_{\mathrm{abs}}$)	Failure under strict native-unit tolerances	LSTM	State-faithful prediction; all models fail (90–100%)
Logged-control inclusion ($m_{\mathrm{incl}}$)	Position of the recorded control sequence among random perturbations	Not used	Auxiliary heuristic only; no ground-truth costs for perturbed candidates
Energy-state composite ($m_{\mathrm{energy}}$)	Speed/altitude/climb RMSE normalized by training-set scales	LSTM	Estimator-loop screening; inspect native-unit channel RMSE alongside
Task proxy ($m_{\mathrm{task}}$)	Downstream screening/tracking cost	Task-dependent	Closed-loop check; tracking exploratory at n=40

**Table 8:** Planning-oriented model selection on 500 in-distribution rollouts. The ranking flips depending on whether models are chosen by local accuracy or by rollout stability. Lower is better in every column.

Model	RMSE@1s	RMSE@20s	Div.@5×	Cap.@5×
MLP	89.14	322.59	90.4%	457.3
TCN	313.38	1235.82	86.0%	453.6
GRU	52.31	64.18	38.6%	210.3
LSTM	**23.88**	**41.20**	52.6%	269.8
Transformer	118.60	88.48	**22.4%**	**157.3**

Note: Bold values indicate the best (lowest) value in each metric column.

**Table 9:** Cross-aircraft transfer to unseen aircraft. Rollout-aware selection remains informative under distribution shift, even though all models degrade in the held-out setting. Held-out values are aggregated over n=300 rollouts on c172r/L410.

Model	RMSE@20s (In)	Div.@5× (In)	RMSE@20s (Held)	Div.@5× (Held)	Cap.@5× (Held)
GRU	64.2	38.6%	319.0	77.0%	392.6
LSTM	41.2	52.6%	353.1	76.3%	386.7
Transformer	88.5	22.4%	275.5	54.3%	302.1

**Table 10:** Sensing-aware open-loop evaluation for the two main competing models on the in-distribution split. RMSE winner remains LSTM in every row, while divergence and the capped-RMSE winner remain Transformer in every row. Lower is better.

Scenario	LSTM RMSE	LSTM Div.	LSTM Cap.	Trans. RMSE	Trans. Div.	Trans. Cap.
Clean	41.2	52.6%	269.8	88.5	22.4%	157.4
Noise-Med	46.9	44.8%	235.4	90.8	21.2%	154.7
Estimated-Med	42.6	51.0%	262.9	89.2	22.4%	157.8
Delay-200 ms	42.0	53.2%	272.8	88.8	22.6%	158.2
Composite-Med	45.7	45.2%	236.0	90.7	23.0%	161.4

**Table 11:** Directly measured shared aggregate-threshold failure rates over 500 validation rollout windows per model. One threshold (5× the cross-model median aggregate RMSE@1s, 445.69) is applied uniformly to the denormalized simulator-coordinate vector. Because coordinates retain heterogeneous units, this is a common-reference benchmark diagnostic rather than a physical tolerance. LSTM is least failing, followed by GRU and Transformer.

Model	Shared Aggregate-Threshold Failure Rate
LSTM	6.6%
GRU	12.8%
Transformer	43.0%

**Table 12:** Descriptive Spearman correlation between open-loop selection metrics and directly simulated downstream outcomes across 18 model/scenario points (GRU, LSTM, and Transformer over five abstract sensing conditions plus the aircraft-specific estimator loop). Positive values indicate that worse open-loop metric values are associated with worse downstream behavior. The points share models, conditions, and source simulations and are not treated as independent inferential samples.

Metric	Tracking Cost	Screening Cost
RMSE@1s	−0.185	−0.279
RMSE@20s	−0.125	−0.255
Div@5×	0.182	0.398
Capped RMSE	0.183	0.402
Physical divergence	−0.194	−0.220

**Table 13:** Trajectory-cluster bootstrap and threshold-sensitivity check for the top three models. Confidence intervals resample 100 source validation trajectories while retaining all five evaluation windows per sampled trajectory under the main 5× criterion. Lower is better in all columns.

Model	Div.@5× [95% CI]	Cap.@5× [95% CI]
Transformer	22.4% [16.4, 28.8]	157.3 [130.3, 186.2]
GRU	38.6% [31.6, 45.8]	210.3 [176.7, 244.6]
LSTM	52.6% [45.2, 59.8]	269.8 [234.2, 305.0]

Threshold sensitivity: under 3×, 5×, and 7× multipliers, the top-three ordering remains Transformer → GRU → LSTM by both divergence rate and capped RMSE.

**Table 14:** Physical absolute-error divergence view for the top three models. Relative divergence uses the self-scaled $5\times \epsilon_{1s}$ benchmark criterion, whereas physical divergence uses strict fixed native-unit stress tolerances. Lower is better in all columns.

Model	Self-Scaled Div@5×	Physical Div	*u* RMSE@20s	Alt. RMSE@20s
Transformer	22.4%	100.0%	21.6 ft/s	291 ft
GRU	38.6%	98.2%	17.9 ft/s	211 ft
LSTM	52.6%	90.4%	11.0 ft/s	136 ft

**Table 15:** Auxiliary logged-control inclusion diagnostic under sensing-aware context corruption. “Position” is the surrogate-assigned position of the logged control sequence among 50 surrogate-scored candidates; higher top-5 inclusion means that sequence appears in the surrogate’s top five more often. Perturbed candidates were not re-simulated in JSBSim, so these values are not ground-truth ranking-fidelity measures and are excluded from model selection.

Scenario	Trans. Position	Trans. Top-5	LSTM Position	LSTM Top-5
Clean	5.15	75%	31.70	0%
Noise-Med	5.80	70%	33.80	0%
Estimated-Med	5.35	70%	33.25	0%
Delay-200 ms	4.95	70%	33.50	0%
Composite-Med	6.10	70%	34.15	0%

**Table 16:** Aircraft-specific estimator-in-the-loop stress test. Planner context is generated from per-aircraft inertial measurement unit (IMU), global navigation satellite system (GNSS), and barometric noise and a lightweight complementary/Kalman-style estimator. Lower is better except success and top-5 rates. Screening under this estimator path favors LSTM, highlighting estimator-path-dependent model choice. The logged-control inclusion rows are auxiliary diagnostics without JSBSim ground truth for perturbed candidates and are not used by the selector.

Metric	LSTM	Transformer	Lower/Higher Value
Open-loop RMSE@20s	55.6	90.3	LSTM
Open-loop Div@5×	66.8%	24.4%	Transformer
Open-loop capped RMSE	339.4	167.0	Transformer
Energy-state $m_{\mathrm{energy}}$	0.557	0.895	LSTM
Aux. logged-control mean position	40.7	3.9	Transformer
Aux. logged-control top-5 inclusion	0.0%	80.0%	Transformer
Tracking task cost	3.197	2.953	Transformer
Tracking success	92.5%	90.0%	LSTM
Screening task cost	5.309	6.027	LSTM
Screening top-5 hit rate	37.5%	5.0%	LSTM

**Table 17:** Sensing-aware downstream assessment for LSTM and Transformer in the abstract sensing-corruption suite. Lower task cost is better. Tracking and screening use 40 episodes each per scenario; small success-rate differences should be interpreted descriptively. For example, Wilson 95% intervals are approximately 76.9–96.0% for 90.0% success and 80.1–97.4% for 92.5% success at this episode count.

Scenario	LSTM Track Cost	LSTM Track Succ.	Trans. Track Cost	Trans. Track Succ.	LSTM Screen Cost	Trans. Screen Cost
Clean	3.151	92.5%	2.925	90.0%	5.084	4.644
Noise-Med	3.197	92.5%	3.055	90.0%	5.086	4.644
Estimated-Med	3.263	92.5%	3.207	90.0%	5.085	4.660
Delay-200 ms	3.533	92.5%	3.345	92.5%	5.057	4.660
Composite-Med	4.052	92.5%	4.079	90.0%	4.977	4.641

**Table 18:** Descriptive consistency of benchmark-specific task heuristics in the abstract sensing-corruption suite. Values are averages over the five sensing-aware scenarios in Table 17; lower cost and higher tracking success are better. Screening and tracking use separately generated task episodes, so this table is not an independently held-out selector validation.

Selector	Screen Model	Screen Cost	Track Model	Track Cost	Track Succ.
RMSE@20s	LSTM	5.058	LSTM	3.439	92.5%
Self-scaled Div.@5×	Transformer	4.650	Transformer	3.322	90.5%
Capped RMSE	Transformer	4.650	Transformer	3.322	90.5%
Physical fidelity	LSTM	5.058	LSTM	3.439	92.5%
Task-conditioned heuristic	Transformer	4.650	LSTM	3.439	92.5%

**Table 19:** Task-conditioned benchmark guidance derived from Table 4. Each row summarizes the reported lexicographic heuristic for a target planning task and estimator path.

Evaluation Use Case	Estimator Path	Heuristic Tuple (from Table 4)	Selection Implication
Candidate screening	Abstract sensing	$\left(m_{\mathrm{task}},\;m_{\mathrm{cap}},\;m_{\mathrm{rel}},\;m_{\mathrm{abs}},\;m_{\mathrm{local}}\right)$	Transformer has lower observed screening cost in the evaluated abstract conditions.
Candidate screening	Estimator loop	$\left(m_{\mathrm{task}},\;m_{\mathrm{energy}},\;m_{\mathrm{cap}},\;m_{\mathrm{rel}},\;m_{\mathrm{abs}}\right)$	LSTM has lower observed estimator-conditioned screening cost.
State-faithful prediction	Any	$\left(m_{\mathrm{abs}},\;m_{\mathrm{energy}},\;m_{\mathrm{local}},\;m_{\mathrm{rel}}\right)$	LSTM preferred for absolute fidelity.
Feedback-rich tracking	Any	$\left(m_{\mathrm{abs}},\;m_{\mathrm{task}},\;m_{\mathrm{energy}},\;m_{\mathrm{local}}\right)$	Endpoints are mixed; reassess in the target controller loop.
Cross-aircraft transfer	Held-out	$\left(m_{\mathrm{abs}},\;m_{\mathrm{cap}},\;m_{\mathrm{rel}},\;m_{\mathrm{local}}\right)$	Rerun on held-out data; do not extrapolate.

**Figure A1:**
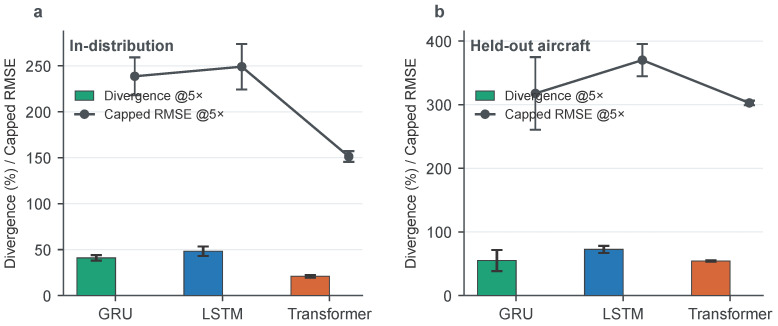
Five-seed variability for the top three models. (**a**) In-distribution five-seed results for GRU, LSTM, and Transformer. (**b**) Held-out-aircraft five-seed results for the same three models. In both panels, bars show divergence rate under the main 5× criterion and black markers show capped RMSE. Error bars denote standard deviation across five seeds (42–46).

## Data Availability

The JSBSim scenario configuration, trained-model evaluation scripts, result metadata, and the evaluation artifacts used for the reported tables will be made available in a public repository upon publication. The accompanying data-generation script now uses deterministic aircraft-specific seeding for future generation. Because the original legacy trajectories used process-dependent seeding, exact bit-for-bit regeneration of those legacy trajectories is not guaranteed; the archived evaluation artifacts provide the provenance for the reported numerical results.

## Abbreviations

The following abbreviations are used in this manuscript:

CI	confidence interval
CPU	central processing unit
CUDA	Compute Unified Device Architecture
EKF	extended Kalman filter
GNSS	global navigation satellite system
GPU	graphics processing unit
GRU	gated recurrent unit
IMU	inertial measurement unit
LSTM	long short-term memory
MAV	micro aerial vehicle
MEMS	microelectromechanical system
MLP	multilayer perceptron
MPC	model-predictive control
OOD	out-of-distribution
RL	reinforcement learning
RMSE	root-mean-square error
RW	random walk
TCN	temporal convolutional network
UAV	unmanned aerial vehicle
UKF	unscented Kalman filter

## Appendix A. Per-Aircraft Sensor and Estimator Parameters

Table A1 reports the exact sensor noise standard deviations and estimator gains used for each aircraft in the estimator-in-the-loop experiment. These values are implemented in the accompanying project code and differ across aircraft to reflect varying sensor quality and estimation dynamics.

**Table A1:** Representative per-aircraft sensor noise and estimator gain parameters used in the aircraft-specific estimator-in-the-loop stress suite. This suite is separate from the five-aircraft surrogate training split; all values are implemented in the accompanying project code.

Parameter	c172p	c172r (Heldout)	f16	L410 (Heldout)
Gyro noise std (rad/s)	0.010	0.011	0.020	0.016
Gyro bias RW sigma (rad/s)	0.0010	0.0010	0.0022	0.0016
GNSS velocity noise (ft/s)	1.2	1.3	3.0	2.3
Barometer noise (ft)	8.0	8.5	16.0	14.0
Vertical-speed noise (ft/s)	1.0	1.0	2.2	1.8
Estimator gain β	0.95	0.95	0.97	0.96
Velocity gain $K_{v}$	0.30	0.29	0.21	0.23
Altitude gain $K_{h}$	0.26	0.25	0.20	0.22
Climb-rate gain $K_{\dot{h}}$	0.32	0.31	0.24	0.27

## Appendix B. Per-Variable RMSE Breakdown

Table A2 reports RMSE at 10 s horizon for the three strongest neural models under state-wise evaluation. Angular rates remain accurate across all models, whereas heading and altitude dominate long-horizon degradation.

**Table A2:** Per-variable RMSE at 10 s horizon.

Variable	GRU	LSTM	Transformer
ψ (rad)	1.168	1.122	1.181
*h* (ft)	163.4	92.7	262.7
*p* (rad/s)	0.018	0.015	0.019
*q* (rad/s)	0.028	0.020	0.046
*r* (rad/s)	0.019	0.015	0.028

## Appendix C. Inference Speed Benchmarks

Table A3 reports mean inference latency for each model versus JSBSim at batch size 1 on a graphics processing unit (GPU) using the Compute Unified Device Architecture (CUDA). All surrogates remain slower than JSBSim at batch size 1 because GPU launch overhead dominates. The motivating use case is therefore large-batch rollout evaluation rather than single-trajectory latency.

**Table A3:** Mean inference latency (ms) at batch size 1 vs. JSBSim on a central processing unit (CPU). Speedup <1 indicates the surrogate is slower than the simulator.

Model	JSBSim@5s (ms)	Surrogate@5s (ms)	Speedup
MLP	4.109	154.592	0.027×
GRU	4.171	103.646	0.040×
LSTM	5.800	188.428	0.031×
TCN	6.205	12.973	0.478×
Transformer	4.887	20.028	0.244×

## Appendix D. Robustness and Negative Results

### Appendix D.1. Noise-Aware Training Is Not Automatically Beneficial

We test whether a naive noise-aware training strategy removes the sensing-evaluation gap. We retrain LSTM and Transformer using medium noisy context inputs and evaluate them on noisy, estimated, and composite sensing conditions.

Table A4 reports the noise-aware training comparison.

**Table A4:** Noise-aware training comparison on sensing-corrupted validation inputs. Each entry reports RMSE@20s/Div@5×/capped RMSE. Lower is better.

Model	Scenario	Clean-Train	Noise-Aware Train	Better
LSTM	Noise-Med	46.9/44.8%/235.4	56.4/58.4%/301.6	Clean
LSTM	Estimated-Med	42.6/51.0%/262.9	53.3/63.0%/322.2	Clean
LSTM	Composite-Med	45.7/45.2%/236.0	56.5/57.4%/296.6	Clean
Transformer	Noise-Med	90.8/21.2%/154.7	102.4/27.0%/183.7	Clean
Transformer	Estimated-Med	89.2/22.4%/157.8	101.3/28.2%/187.8	Clean
Transformer	Composite-Med	90.7/23.0%/161.4	103.5/33.6%/210.7	Clean

The comparison shows a negative but useful result: simple corruption augmentation is not enough. For both LSTM and Transformer, training on medium-noise contexts worsens RMSE@20s, divergence, and capped RMSE across all evaluated sensing scenarios. We hypothesize that injecting i.i.d. noise into context states during training increases gradient variance and disrupts the hidden-state dynamics that recurrent models rely on for long-horizon stability; the training distribution mismatch between raw injected noise and the smoother estimator-filtered inputs seen at test time may further widen this gap. Confirming this mechanism is left for future work. This means that sensing-aware surrogate selection cannot be reduced to “add noise during training”; the selection problem remains real, and robust sensing-aware training likely requires structured noise models or estimator-aware data augmentation rather than naive context corruption.

### Appendix D.2. Five-Seed Robustness Preserves the Main In-Distribution Winner Frequencies

Figure A1 adds an explicit training-stochasticity check to the rollout-bootstrap intervals. We further extend this check to all five main architectures over five seeds. In-distribution, the winner frequencies are fully stable: LSTM wins RMSE@20s in 5/5 seeds, while Transformer wins both self-scaled Div@5× and capped RMSE in 5/5 seeds. The multi-seed extension requested batch size 512 with automatic mixed precision for newly generated checkpoints, while seed 42 reuses the legacy base checkpoint used throughout the main paper. Because the legacy result metadata do not record an identical batch/precision provenance for every seed, this analysis supports winner-frequency robustness rather than a strictly controlled hyperparameter-identical replication. Held-out results are less stable, which is itself informative: Transformer wins held-out RMSE@20s in 3/5 seeds and capped RMSE in 4/5 seeds, whereas held-out relative-divergence winners are split across Transformer, GRU, and MLP. This broader multi-seed sweep therefore supports a narrower claim than the earlier single-run story: the in-distribution ranking disagreement is robust, but transfer rankings should be interpreted only within the tested JSBSim aircraft set and with explicit uncertainty.

## Appendix E. Training Hyperparameters

Table A5 summarizes the training and robustness-run hyperparameters.

**Table A5:** Main and robustness-run hyperparameters. The five-seed extension reuses the legacy seed-42 base checkpoints and generates missing seeds with the reported extension settings; it is not claimed as a hyperparameter-identical replication across all five seeds.

Hyperparameter	Value
Optimizer	Adam
Learning rate	$10^{-3}$
Epochs	50
Main base-checkpoint batch size	256
Multi-seed extension requested batch size	512 (automatic mixed precision enabled for generated runs)
Context window $T_{c}$	50 steps (5 s)
Evaluation rollout horizon	200 steps (20 s)
Training aircraft	c172p, c182, c310, pa28, DHC6
Held-out aircraft	c172r, L410
Main seed count	5
Multi-step loss horizons	10, 50, 100, 200 steps
